# The Dual Role of Antimicrobial Proteins and Peptides: Exploring Their Direct Impact and Plant Defense-Enhancing Abilities

**DOI:** 10.3390/plants13152059

**Published:** 2024-07-26

**Authors:** Atefeh Farvardin, Ana Isabel González-Hernández, Eugenio Llorens, Gemma Camañes, Loredana Scalschi, Begonya Vicedo

**Affiliations:** 1 Biochemistry and Biotechnology Group, Department of Biology, Biochemistry and Natural Sciences, Universitat Jaume I, 12071 Castellón de la Plana, Spain; farvardi@uji.es (A.F.); camanes@uji.es (G.C.); bvicedo@uji.es (B.V.); 2 Superior Polytechnic School of Zamora, University of Salamanca, Avda. de Requejo, 33, 49029 Zamora, Spain; aigonzalez@usal.es

**Keywords:** plant APPs, plant defense, biotic stress, abiotic stress, natural treatment, sustainable agriculture

## Abstract

Plants face numerous environmental stresses that hinder their growth and productivity, including biotic agents, such as herbivores and parasitic microorganisms, as well as abiotic factors, such as cold, drought, salinity, and high temperature. To counter these challenges, plants have developed a range of defense strategies. Among these, plant antimicrobial proteins and peptides (APPs) have emerged as a promising solution. Due to their broad-spectrum activity, structural stability, and diverse mechanisms of action, APPs serve as powerful tools to complement and enhance conventional agricultural methods, significantly boosting plant defense and productivity. This review focuses on different studies on APPs, emphasizing their crucial role in combating plant pathogens and enhancing plant resilience against both biotic and abiotic stresses. Beginning with in vitro studies, we explore how APPs combat various plant pathogens. We then delve into the defense mechanisms triggered by APPs against biotic stress, showcasing their effectiveness against bacterial and fungal diseases. Additionally, we highlight the role of APPs in mitigating the abiotic challenges associated with climatic change. Finally, we discuss the current applications of APPs in agriculture, emphasizing their potential for sustainable agricultural practices and the need for future research in this area.

## 1. Introduction

**Figure 1:**
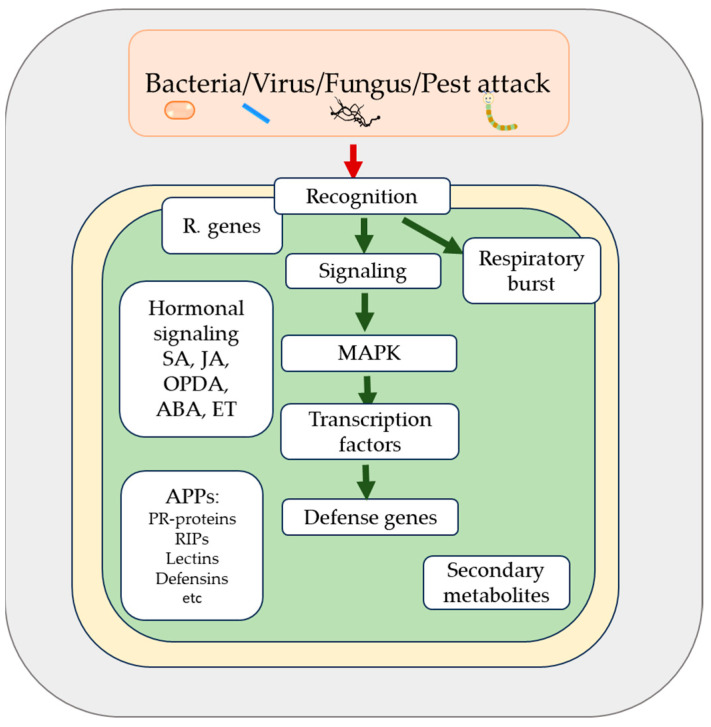
Schematic representation of the plant defense mechanism to different types of plant attackers. The figure illustrates the pivotal roles of key signaling molecules such as MAPK (mitogen-activated protein kinases) and hormonal pathways in the intricate mechanisms of plant defense. Hormonal pathways include SA (salicylic acid), JA (jasmonic acid), OPDA (12-oxo-phytodienoic acid), ABA (abscisic acid), and ET (ethylene). Additionally, it highlights the crucial participation of antimicrobial proteins and peptides (APPs) such as PR proteins (pathogenesis-related proteins), RIPs (ribosome-inactivating proteins), lectins, defensins, and other essential components involved in the plant’s immune responses.

Plants are sessile organisms and must contend with a variety of pathogens and environmental stress factors. The reliance on chemical pesticides to combat plant diseases, insect pests, and abiotic stresses has raised concerns due to their potential harm to humans, animals, and the environment. Plant defense mechanisms are complex and encompass intricate networks. The first mechanism of defense is the physical barrier composed of the cell wall, waxy cuticle, and trichomes, which restrains pathogen entry into the plant cells [1,2]. Once the pathogen overcomes this first barrier, plant cells are capable of recognizing threats and eliciting effective defense responses, which are commonly known as the plant immune system [3,4,5]. Plant immunity studies have shown that plant transmembrane pattern-recognition receptors (PRRs) are responsible for detecting microbial-associated molecular patterns (MAMPs) and initiating a quick basal resistance upon detecting the stressor, like hypersensitive response [6,7]. However, harmful microorganisms have developed different mechanisms to overcome the immunity triggered by MAMPs, while plants have also evolved. Antimicrobial proteins and peptides (APPs) are essential in plant immunity, offering robust resistance against biotic stress and showing significant potential in agronomic, food industry, medical, and pharmaceutical fields [7,8]. The diversity and versatility of these defense responses leads to the induction of different antimicrobial peptides and proteins. Recent research has shown that APPs genes are integrated into the plant immunity-signaling network, with their expression being regulated by the same core signaling module that controls various other plant defense responses. Plant hormones such as jasmonic acid, ethylene, and salicylic acid, which are central regulators of plant immune responses, are frequently reported as strong up-regulators of APP gene expression across numerous plant species. These hormones modulate transcription factors that are crucial for APP responses during pathogen attacks. The connection between defense hormones and APP induction is so prominent that certain APP genes are recognized as key markers for studying the activation of plant hormonal and immune system signaling pathways [9,10] (Figure 1).

The majority of the natural APPs are composed of 10 to 50 amino acids, ranging from 2 to 9 kDa, and they can be divided into anionic and cationic peptides depending on the electrical charge [11]. APPs have been isolated from different plant tissues (roots, seeds, flowers, stems, and leaves) from a diverse array of plant species [12]. Their application has demonstrated efficacy against a wide range of phytopathogens and human pathogens, including viruses, bacteria, fungi, and protozoa [13,14]. Previous studies have shown that APPs not only rupture lipid cell membranes but also enter the cytoplasm to disrupt cellular physiological processes, inducing a reduction in pathogen cell growth and cell death [15,16]. APPs induce phytohormone genes such as salicylic acid (SA), jasmonate-dependent pathways, and R-gene signaling [17]. Furthermore, the defense strategy is bolstered by the fact that transgenic plants overexpressing antimicrobial peptide and protein genes confer resistance to bacterial and fungal attack in different plant species [18,19]. However, their efficacy depends on the APP concentration and the pathogen density [20]. The most important families of APPs are pathogenesis-related (PR) proteins, ribosome-inactivating proteins (RIPs), protease inhibitors (PIs), heme-binding proteins, lectins, defensins, lipid transfer proteins (LTPs), thionins, snakins/GASA (giberellic acid stimulated in Arabidopsis), cyclotides, and hevein-like proteins [21,22,23]. Nevertheless, it should be noted that the sequence of plant APPs is highly variable, so it is crucial to study their characteristics to delve deeper into the action mechanisms.

As previously described by Tang et al. [8], the advantages of the application of APPs to crops are the fast fungal and bacterial killing effect, the ease of integration into transgenic transformation processes, the synergy with other antimicrobial agents, the manipulation of the symbionts, and plant growth promotion.

Nevertheless, the disadvantages are the low systemic effect, the high required APP concentration, the complexity of purification, the expensive production, and the selection of resistant pathogens. Large-scale field investigations and uses of APPs are currently rare due to their high production costs, but researchers are working on different systems to produce APPs cheaply [24,25]. Hence, APPs constitute an alternative to the commonly used antimicrobial agents in agriculture due to their broad-spectrum antimicrobial activity, killing potential, high selectivity, and low toxicity. However, further understanding is required to afford the mentioned disadvantages.

The aim of this review is to explore the diversity and functionality of proteins and peptides in plant defense, covering well-known APPs. Furthermore, a summary of the molecular mechanisms, the involved signaling pathways, and the potential of these molecules in developing sustainable crop protection strategies will be addressed. Thus, this review will not only provide a detailed understanding of how plants use these components to protect themselves but also highlight emerging biotechnological innovations that could revolutionize plant defense and enhance agricultural resilience against biotic and abiotic challenges.

## 2. Plant-Derived APPs

APPs are fundamental elements of plant defense, constituting a frontline defense mechanism against pathogens [26]. They showcase innate immunity, swiftly countering invaders through a repertoire of mechanisms.

APPs were predominantly extracted from seeds but also from various other plant parts, exhibiting activity against phytopathogens [11,27]. Their primary action involves membrane disruption, alongside interference with intracellular processes such as DNA and protein synthesis [28]. The composition and structure of these peptides vary depending on the plant species and the pathogens they encounter. APPs are found in all plants, showing molecular diversity within species, and are crucial for defending against biotic stress [29].

Categorically, plant APPs are classified into different families based on structural similarities, with some families widely conserved and others specific to plants or individual species. They are further categorized by size into two groups: large antimicrobial proteins like chitinases and glucanases, activated in response to fungal attacks, and smaller proteins known as antimicrobial peptides (AMPs) [22]. Antimicrobial proteins include PR proteins, RIPs, PIs, heme-binding proteins and lectins. PR proteins induced upon pathogen attack include PR-1 with antifungal activity [30], PR-2 like β-1,3-glucanases degrading fungal cell walls and showing antibacterial activity [31], and PR-3, PR-4, PR-8, and PR-11, such as chitinases breaking down fungal cell wall chitin [14,32]. PR-5, a thaumatin-like protein, disrupts fungal cell wall integrity while PR-6 functions as a protease inhibitor and causes excision of exopeptidases produced by fungus, bacteria, and insects [33]. RIPs are toxic enzymes found in many plants. They disrupt protein synthesis by removing purine bases from rRNA in both eukaryotes and prokaryotes. RIPs have been shown to confer protection against fungi, bacteria, viruses, and insects, both in vitro and in genetically modified plants [34]. PIs shield plant proteins from degradation by inhibiting pathogen proteases, while lectins bind to microbial surfaces, agglutinating and hindering bacterial and fungal growth [23]. Recently, an apoplastic heme-binding protein with antimicrobial properties has been identified which acts by disrupting bacterial cell walls, leading to leakage of intracellular contents [22].

Antimicrobial peptide families include thionins (PR13 family), defensins (PR12 family), LTPs (PR14 family), hevein-like peptides, knottin-type peptides, α-hairpinins, snakins, and cyclotides. These peptides typically possess positive charges and amphiphilic properties, facilitating direct interactions with microbial membranes. Additionally, they are stabilized by disulfide bonds, enhancing their structural integrity and antimicrobial efficacy.

Thionins, hailed as groundbreaking plant antimicrobial peptides, have demonstrated remarkable efficacy against plant pathogens in vitro, earning the distinction of being the first eukaryotic peptides recognized for their defensive capabilities. Their consistent antimicrobial activity spans a wide spectrum of phytopathogenic bacteria and fungi.

Meanwhile, defensins represent another formidable line of defense among plant AMPs. These compact, cysteine-rich peptides are ubiquitously distributed throughout the plant kingdom, playing a pivotal role in fortifying plant immunity against microbial invaders [35,36].

Among the intriguing discoveries are the snakins/GASA family, belonging to the cysteine-rich peptide (CRP) group. They can inhibit a wide range of bacterial and fungal growth at extremely low concentrations [37]. Skanins derived from potato tubers include cell wall-associated peptides like snakin-1 (StSN-1) and snakin-2 (StSN-2) [38]. GASA peptides, another member of this family, are widely distributed across different plant species, showing organ-specific and developmental stage-specific expression. Although GASA’s subcellular localization varies, it is primarily found in the cell wall and is regulated by gibberellic acid (GA) [39].

Additionally, LTPs have been uncovered in various plant species, ranging from radish to onion. These proteins exhibit varying degrees of efficacy in inhibiting bacterial and fungal pathogens [40].

Moreover, cyclotides, found within diverse plant families including Cucurbitaceae, Fabaceae, Violaceae, and Rubiaceae, exhibit a captivating array of biological activities, including potent antimicrobial properties.

**Figure 2:**
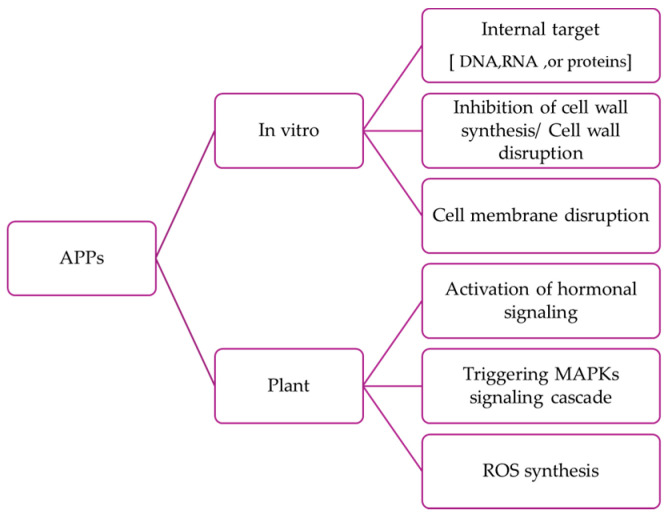
Schematic diagram of the mode of action of APPs. It includes both in vitro and in-plant mechanisms.

Mechanistically, APPs vary in action; some disrupt microbial cell wall/membranes causing lysis, while others penetrate microbial cells to disrupt essential intracellular components like DNA, RNA, or proteins. Additionally, beyond their antimicrobial functions, these molecules coordinate plant defense responses against diverse stresses, including biotic and abiotic challenges (Figure 2) [24]. Therefore, the wide variability of APPs enables an adaptive response to various pathogens [7] as demonstrated by the use of transgenic plants [41,42,43,44,45]. Although the primary function of APPs is to combat microbial pathogens, their influence extends to a wide range of plant attackers. These include Gram-positive and Gram-negative bacteria, phytopathogenic fungi and oomycetes with varying lifestyles, nematodes, mollusks, piercing-sucking insects like aphids, leaf-chewing insects, and even the parasitic plant *Orobanche cumana* [7].

Through intricate molecular mechanisms, they orchestrate the activation of diverse signaling pathways, including those governed by salicylic acid, jasmonates, R-genes, and mitogen-activated protein kinases (MAPKs). Moreover, they serve as catalysts for the production of reactive oxygen species (ROS), contributing significantly to the overall resilience of plants against the complexities of their environment [46].

While over 3000 experimentally confirmed APPs have been identified, ongoing research efforts continue to unveil novel variants of these molecules, shedding light on their distribution patterns and functional diversity across different plant species. However, elucidating the intricate interplay between APPs and the regulatory networks governing their expression and activation within plant genomes poses a complex scientific challenge that warrants further investigation. A summary of the different families of plant APPs and their functions can be found in Table 1.

## 3. Exploring the Antimicrobial Potential of Plant APPs In Vitro

The in vitro study of APPs enables the selection of active molecules against pathogens for plant application. Thionins, for instance, are AMPs exclusively found in plants and have demonstrated potential in controlling such diseases. Thionins have repeatedly been shown to have antimicrobial activities in vitro against different phytopathogenic bacteria and fungi.

Another group of well-known antimicrobial plant peptides is the defensins. One example is the alfalfa defensin (alfAFP), extracted from *Medicago sativa* seeds which exhibited potent activity against the agronomically important fungal pathogen *Verticillium dahliae* [68]. Additionally, this defensin demonstrated inhibitory effects on the growth of other fungal plant pathogens like *Alternaria solani* and *Fusarium culmorum*. Moreover, Rs-AFP2, a plant defensin from radish, was shown to inhibit the growth of several fungi [69]. The antimicrobial activity of NmDef02, a defensin isolated from *Nicotiana megalosiphon* upon inoculation with the tobacco blue mold pathogen *Peronospora hyoscyami* subsp. *tabacina* was also investigated [70]. The recombinant NmDef02 defensin exhibited antimicrobial activity against several important common plant pathogens: *Phytophtora infestans*, *Phytophtora parasitica* var. nicotianae, *A. solani*, *Fusarium oxysporum* var. cubense and *V. dahliae*. A small cysteine-rich protein, designated as a defensin (SPD1), was isolated from sweet potato storage roots. It has been found to inhibit the growth of both fungi and bacteria. Notably, SPD1 represents the first reported plant defensin to exhibit dehydroascorbic acid reductase (DHA) and monodehydroascorbate reductase (MDA) activities.

Peptides called snakins have been isolated from potato tubers. They comprise the cell wall-associated peptide snakin-1 (StSN-1) and snakin-2 (StSN-2). Segura et al. [63] successfully demonstrated StSN-1 antimicrobial activity against bacterial and fungal plant pathogens such as *Clavibacter michiganensis* subsp. *sepedonicus* and *B. cinerea*, respectively. Moreover, the anti-yeast potential of this peptide was tested against a number of common food spoilage yeasts [71]. The StSN-2 peptide is also active in vitro against bacterial and fungal plant pathogens. It causes a rapid aggregation of both Gram-positive and Gram-negative bacteria, although this property did not correlate with its inhibitory activity. 

Two antimicrobial peptides, Ac-AMP1 and Ac-AMP2, which share similarities with chitin-binding proteins, were isolated from amaranth seeds *(Amaranthus caudatus*). These peptides demonstrated antimicrobial efficacy against both Gram-positive bacteria and plant pathogenic fungi. Interestingly, their antimicrobial function was counteracted by cations [72].

Plant LTPs inhibit the growth of bacterial and fungal pathogens to different degrees. The LTP isolated from onion seeds (Ace-AMP1) exhibited a potent effect in vitro since it was able to inhibit the growth of 12 tested fungi and Gram-positive bacteria at concentrations below 10 μg/mL [73]. This LTP isolated from onion seeds exhibited a higher antimicrobial activity than the LTP extracted from radish seeds. The antifungal activity of an LTP isolated from sunflower (*Helianthus annuus*) seeds was reported by Regente and Canal [74].

Cyclotides have been discovered to possess antimicrobial properties. They exhibit strong activity against model fungal plant pathogens like *F. oxysporum*, *F. graminearum*, *F. culmorum*, *Mycosphaerella fragariae*, and *B. cinerea*, and also against phytopathogenic bacteria, such as *Pseudomonas syringae* pv. syringae, *Dickeya dadantii*, and *Pectobacterium atrosepticum* [75].

A recent study revealed the 3D structure of NCR044, a 36-amino acid antimicrobial peptide present in the nodules of the model legume *Medicago truncatula* [62]. This peptide, characterized by its cysteine-rich composition, exhibited a potent antimicrobial effect against various fungal pathogens, notably *B. cinerea* and three species of *Fusarium*. NCR044 demonstrated inhibitory effects on spore germination of *B. cinerea*. Moreover, upon penetrating the cell membrane of germinating spores, the peptide accumulated within the cytoplasm and nucleoli, triggering the generation of ROS.

Citrus Huanglongbing (HLB), also known as citrus greening, is caused by the vector-transmitted phloem-limited bacterium *Candidatus* Liberibacter asiaticus (*C*Las) and is the most destructive disease-threatening citrus in industries worldwide. Huang et al. [76] characterized a heat-stable antimicrobial peptide MaSAMP from HLB-tolerant *Microcitrus australiasica*. This peptide can rapidly kill *Liberibacter crescens* (*Lcr*), a culturable Liberibacter strain.

Additionally, in the context of antimicrobial proteins, a study demonstrated that Alpha-momorcharin (α-MMC), a RIP purified from the seeds of *Momordica charantia*, showed a potent inhibitory effect on the growth of several fungal pathogens, including *Bipolaris maydis*, *A. niger*, *Aspergillus oryzae*, *F. graminearum*, and *S. sclerotiorum*. Among these, *B. maydis* was the most significantly inhibited by α-MMC [77]. Similarly, another study found that crude lectin from Moringa seeds was highly effective in inhibiting the growth of *B. cinerea*, *Corynespora cassiicola*, and *A. alternata* [78]. In vitro experiments conducted by Carillo et al. [79] demonstrated that barley cysteine and serine PIs effectively inhibited fungal growth. However, these PIs did not show any significant effect on bacterial growth. Furthermore, an in vitro analysis has shown that the *Solanum lycopersicum* heme-binding protein (SlHBP2), an apoplastic protein extracted from tomato plants treated with 1-Methyl tryptophan, effectively combats several plant pathogens, notably *P. syringae* [22].

## 4. Exploring the Role of APPs in Plants

### 4.1. Empowering Plants with APPs to Manage Biotic Stresses

Research into plant-based antimicrobials has uncovered promising avenues for enhancing plant defenses against various pathogens. Niu et al. [80] showcased the effectiveness of *Capsicum annuum* antimicrobial protein 1 (CaAMP1) in bolstering soybean resilience against *Phytophthora* root rot. By overexpressing *CaAMP1*, soybeans exhibited robust resistance responses. Similarly, another study demonstrated that transgenic potatoes overexpressing a barley-derived chitinase gene displayed strong resistance to *A. solani*. These transgenic potato plants remained green and healthy post-infection, while non-transgenic plants turned yellow and died [81].

In line with this, Rode et al. [82] provided further insights into plant defense mechanisms by demonstrating the efficacy of Lipid Transfer Protein1 (LTP1) in controlling *Xanthomonas oryzae* infection in rice. They also introduced exogenous *Citrus sinensis* LTP1 (CsLTP1) treatment, uncovering activated phytochemicals and metabolic pathways. Su et al. [83] contributed by revealing the effectiveness of the proline-rich protein (PnPRPL1) recombinant protein in suppressing root rot pathogens’ growth and conidial germination, identifying its regulation by a WRKY transcription factor.

Innovative approaches, exemplified by Beliaev et al. [67], showcased the use of the *proSmAMP1* gene from chickweed, which encodes two hevein-like peptides to enhance potato resistance against early blight, yielding promising results in commercial potato varieties. Lacerda et al. [84] demonstrated that the recombinant pea defensin Drr230a, referred to as rDrr230a, significantly reduced the severity of Asian soybean rust, further supporting the potential of plant-based antimicrobials. Likewise, AtPep1, a 23-aa peptide from Arabidopsis, has been found to trigger the activation of the defensive gene defensin (*PDF1.2*) and H_2_O_2_ synthesis. By overexpressing the AtPep1 precursor gene, *PROPEP1*, root development and resistance to the pathogen *Pythium irregulare* are enhanced [85].

In a separate study focusing on defensin efficacy, researchers discovered that the expression of apoplast-targeted plant defensin MtDEF4.2 in transgenic wheat provides substantial resistance against a prevalent leaf rust disease. Notably, this defense mechanism operates without compromising the beneficial symbiotic relationship with mycorrhizal fungi [86]. Additionally, defensin treatment extracted from *Trigonella foenum-graecum*, significantly boosts tomato plant resistance to bacterial wilt, improving water content, gas exchange, and nutrient uptake while reducing disease severity and oxidative stress in hydroponic culture [87]. Stotz et al. [88] showcase the broad impacts of tomato defensin DEF2 overexpression on foliar resistance to *B. cinerea*.

Moreover, according to Hao et al. [89], overexpression of a modified plant thionin improves resistance to citrus canker. Additionally, the successful integration of thionin genes into potato plants led to enhanced resistance against *B. cinerea* [90].

Furthermore, Hsiang-En Huang et al. [91] highlighted the efficacy of recombinant plant Ferredoxin-like Protein (PFLP) in reducing soft-rot symptoms in tobacco plants infected by *Erwinia carotovora*.

Also, Gully et al. [92] discovered that applying synthetic SCOOP12, derived from a family of 14 Arabidopsis genes producing serine-rich endogenous peptide precursors (PROSCOOP), triggers various defense mechanisms in Arabidopsis. Transcriptomic analysis showed that PROSCOOP12 is associated with responses to biotic and oxidative stress and influences root growth. Their research indicates that SCOOP12 exhibits several characteristics of phytocytokines, activates the phospholipid signaling pathway, modulates the ROS response, and operates through a BAK1 co-receptor-dependent mechanism.

In a similar context, another study highlights the importance of Gibberellin Stimulated-Like proteins, specifically GSL2 (also known as Snakin-2) from potato (*Solanum tuberosum* L.), in plant defense. Overexpression of the *GSL2* gene in transgenic potatoes has been shown to confer resistance to blackleg disease caused by *P. atrosepticum.* This finding confirms that GSL2 plays a crucial role in enhancing the plant’s defense mechanisms against pathogens [42].

Lastly, Morais et al. [93] investigated a chimeric protein SlP14a-PPC20 obtained by linking PPC20, a peptide derived from sunflower phosphoenolpyruvate carboxylase to SlP14a, a *S. lycopersicum* pathogenesis-related protein. This protein showed potency in combating bacterial wilt disease in tomatoes caused by *Ralstonia solanacearum*, demonstrating its effectiveness in reducing bacterial populations and disease severity.

As previously mentioned, MaSAMP was discovered in the HLB-tolerant Australian finger lime (*Microcitrus australasica*), a close relative of citrus. SAMP not only significantly lowered the titer of *C*Las and alleviated disease symptoms in HLB-positive trees but also activated innate immunity, thereby preventing and inhibiting infection [76].

These studies collectively emphasize the significant role of plant-based antimicrobials in enhancing plant defenses against diverse pathogens, offering novel strategies for improving crop resilience.

### 4.2. Enhancing Plant Defense against Abiotic Stress with APPs

Abiotic stresses present significant challenges to crop production, with extreme temperatures, salinity, drought, and flooding emerging as major factors contributing to yield reduction. Recent research has shed light on novel approaches to enhance plant defense mechanisms against abiotic stresses. Wytynck et al. [94] reported that transgenic rice lines overexpressing RIPs, specifically OsRIP1 and nuRIP, affect methyl jasmonate-mediated stress responses. Furthermore, Tawari et al. [95] reported that the OCPI2 gene, which belongs to the rice chymotrypsin protease inhibitor gene family, is a promising candidate for genetic enhancement to improve plant resistance to salt and osmotic stress. Cirillo et al. [61] demonstrated that treating tomato plants with a picomolar solution of systemin significantly bolstered their resilience to salt stress by activating key sodium ion transporters (SOS1, NHX, and HKT) in the leaves, thereby bolstering the cellular antioxidant capacity, and maintaining balanced protease activity despite the stress. In parallel, Criscuolo et al. [96] found that N-terminal protein fragments of Prosystemin help alleviate salt stress in tomato plants. This effect is linked to the upregulation of crucial stress-related genes (*CAT2*, *APX2*, and *HSP90*), which enhance antioxidant activity and free radical scavenging in stressed plant cells.

Similarly, Bashir et al. [97] highlighted the importance of osmotin, a stress-responsive protein belonging to the pathogenesis-related 5 (PR-5) family, in combating various environmental stressors. Osmotin aids in inducing osmo-tolerance by regulating proline content, maintaining osmotic balance, and protecting cellular components. It plays a crucial role in enhancing plant resilience to salinity stress by removing sodium ions and controlling hydrogen peroxide accumulation. Additionally, osmotin contributes to cold and heat tolerance by regulating cellular processes and activating signal pathways that enhance thermotolerance.

In the ongoing pursuit of using plant APPs to combat abiotic stress, Tian et al. [98] demonstrated the efficacy of TaCEP1D peptide encoded by TraesCS1D02G130700 in enhancing drought tolerance in wheat plants through the mitigation of ROS accumulation and lipid peroxidation.

Concurrently, Faragó et al. [99] investigated the role of small paraquat resistance proteins (SPQ) in enhancing drought tolerance in Arabidopsis plants. Their findings revealed that overexpressing SPQ enhances plant viability and sustains photosynthetic activity during drought conditions. This improvement is achieved by affecting the plant’s sensitivity to abscisic acid and its responses to oxidative stress.

Expanding the scope, Liu et al. [48] showcased the potential of PR proteins with chitinase activity, such as LcCHI2 from *Leymus chinensis*, in enhancing salt tolerance in *Nicotiana tabacum* and *Zea mays*. While promising, further research is needed to elucidate the precise mechanisms underlying this salt tolerance enhancement.

While the use of APPs in water-deficit stress and metal toxicity is limited, Kumar et al. [56] revealed a promising breakthrough. Their study showed that overexpressing the Chickpea defensin gene (*Ca-AFP*) in *Arabidopsis thaliana* significantly enhances tolerance to water-deficit stress. The transgenic plants exhibited heightened activities of crucial antioxidant enzymes—superoxide dismutase, ascorbate peroxidase, and catalase. Moreover, they displayed increased levels of proline and chlorophyll, along with higher relative water content. Remarkably, these plants exhibited reduced ion leakage and malondialdehyde content under water-deficit conditions, indicating the potential efficacy of Ca-AFP in bolstering plant resilience to water scarcity.

Moreover, Liu et al. [100] highlight the role of the plant defensin-dissimilar thionin OsThi9 in alleviating cadmium toxicity in rice plants, leading to reduced cadmium accumulation in rice grains.

Furthermore, Wang et al. [39] showed that expressing *Salvia miltiorrhiza SmGASA4* in Arabidopsis enhances flower and root development and increases the plant’s resistance to salt, drought, and paclobutrazol (PBZ) stress.

Contrary to previous findings, Xu et al. [101] have identified a novel mitochondria-localized cysteine-rich transmembrane module (CYSTM) member, termed CYSTM3, as a negative regulator of salt stress tolerance in Arabidopsis. Their study reveals that CYSTM3 functions as a negative regulator by impeding Na^+^ efflux and disrupting reactive oxygen species’ (ROS) homeostasis.

Another opposing effect of APPs was observed in the case of GASA peptides, specifically GASA5, since the overexpression of this peptide in *A. thaliana* increased plant sensitivity to heat stress. This heightened sensitivity was accompanied by decreased expression of genes encoding heat-shock proteins and elevated accumulation of H_2_O_2_ [102].

These studies collectively highlight the potential of applying antimicrobial proteins, peptides, and stress-responsive genes to enhance plant defense mechanisms against a broad spectrum of abiotic stresses, offering promising avenues for enhancing crop resilience and productivity in the face of changing environmental conditions.

## 5. APPs: The Future of Agriculture

Globally, crop yield losses due to plant pathogens and pests can soar as high as 40%, a staggering figure that far surpasses the modest 1–3% annual yield gains achieved through advanced breeding technologies [8]. To counteract this substantial threat to agricultural productivity, it is imperative to focus on preventing these losses. Eco-friendly antimicrobial compounds offer promising avenues for safeguarding crops while minimizing environmental harm.

There are now more than 1700 known natural AMPs. Many derivatives and analogs have been developed, either through computational methods or by synthetic manufacturing, using these natural AMPs as templates [103].

Specifically, plant APPs emerge as sustainable alternatives to conventional agrochemicals, showcasing multiple advantages such as antimicrobial activity, stimulation of plant defenses and growth, and attraction of natural enemies of herbivorous insects. Consequently, there is a growing emphasis on researching these natural compounds to devise effective, sustainable solutions for managing pests and diseases in agriculture.

The AMPs with agricultural activity are reviewed in detail by Zhang et al. [104]. To date, 18 peptides of different origins have been commercialized as green agents for plant protection [104]. The use of the bacterial harpin protein was the first example of the utilization of the strategy of priming plant immunity as a commercial disease prevention strategy [105]. The peptide maSAMP is one of the plant-derived peptides explored as a commercial immune inducer. As mentioned, it has an antibacterial effect and also activates the plant immune system to prevent subsequent infection.

Most agricultural antibiotics and fungicides only target pathogens during their active growth phases, leaving dormant spores unaffected. Recent research, however, shows that AMPs can kill non-germinating fungal spores, significantly reducing pathogen transmission. This is particularly beneficial in greenhouses, where airborne spores cause plant diseases and health risks for workers. Acting as sporicides, AMPs help prevent disease spread and protect workers’ health, and they can also enhance the effectiveness of other antimicrobial treatments.

Moreover, as mentioned earlier in this review, APPs play a vital role in enabling plants to endure harsh environmental conditions like extreme temperatures, drought, and salinity by enhancing the plant’s defense mechanisms. Thus, APPs become invaluable assets in sustainable agriculture. In a world where climate variability presents substantial obstacles to crop production, APPs offer a promising solution for improving resilience and ensuring food security.

## 6. Conclusions and Outlook

Agriculture is currently facing significant challenges due to global climate change and the rise of harmful pathogens, which existing resources struggle to control. Plants naturally use specific proteins and peptides to defend against these stresses. Research into these molecules’ structure and function offers potential solutions to the problems affecting food production.

Given these challenges, the widespread use of chemical antimicrobials in agriculture has raised concerns about health risks and antibiotic resistance, driving demand for natural alternatives. This demand is expanding the market for plant antimicrobial proteins and peptides, preferred for their high efficacy, low toxicity, and environmental safety.

Extensive research into the application, production, and discovery of agricultural peptides is advancing the field of peptide agrochemicals. Biotechnology and genetic engineering have enabled the development of highly effective plant-based peptides for farming. Current research on the effects of specific peptides during abiotic stress conditions in plants has demonstrated their potential in alleviating stress linked to climate change. This promising avenue offers strategies to mitigate these challenges, potentially reducing substantial losses in agricultural and food production caused by such environmental conditions.

As awareness of the health benefits of natural antimicrobials increases and concerns about antibiotic resistance grow, the market for plant antimicrobial proteins and peptides is poised for significant growth. These developments offer sustainable solutions to contemporary agricultural challenges, promoting a safer and more resilient food production system.

## Figures and Tables

**Table 1:** List of plant families of APPs and their respective function.

Antimicrobial Proteins			
Families	Example	Combating Pathogens, Insects, and Abiotic Stressors	Reference
Pathogenesis-related proteins	*AtPR1*	*Sclerotinia sclerotiorum*	[47]
	*LcCHI2* (encodes a class II chitinase)	Salt stress tolerance	[48]
Ribosome-inactivating proteins	PhRIP I	*Botrytis cinerea* *Rhizoctonia solani*	[34]
	OSRIP18	Drought tolerance Salt tolerance	[49]
	Quinoin	*Cryphonectria parasitica*	[50]
Protease inhibitors	Oryzacystatin 1	Drought stress tolerance	[51]
	Psc-AFP	*Ralstonia solanacearum* *Alternaria. alternata*	[52]
Heme-binding proteins	SlHBP2	*Pseudomonas syringae* pv. *tomato* *Botrytis cinerea* *Xanthomonas vesicatoria*	[22]
Lectins	Palectin 16	Salt stress tolerance	[53]
	chickpea Lectin	*Alternaria brassicae* Salt stress tolerance Drought stress tolerance	[54]
Thionins	Thio-60	*Fusarium oxysporum*	[55]
Defensins	Ca-AFP	Water-deficit stress	[56]
	MsDef1	*Xanthomonas campestris* *Ralstonia solanacearum* *Xanthomonas campestris* *Aspergillus niger* *Pyricularia oryzae* *Rhizoctonia solani* *Pseudomonassyringae* pv. *tabaci*	[57]
Lipid transfer Proteins	NtLTP4	Salt stress tolerance Drought stress tolerance	[58]
	TdLTP4	*Alternaria solani* *Botrytis cinerea*	[59]
Systemin	Systemin	*Botrytis cinerea*	[60]
	Systemin	Salt stress tolerance	[61]
Nodule-specific cysteine-rich (NCR) peptides	NCR044	*Botrytis cinerea* *Fusarium graminearum* *Fusarium virguliforme*	[62]
Snakins/GASA family	Snakin-1	*Clavibacter michiganensis* subsp. *sepedonicus* *Botrytis cinerea*	[63]
	Snakin-2	*Clavibacter michiganenesis* subsp. *sepedonicus* *Botrytis cinerea*	[64]
	AtGASA14	Salt stress tolerance	[65]
Cyclotides	kalata B1, and kalata B2	*Pomacea canaliculata*	[66]
Hevin-like proteins	Pro-SmAmp1	*Alternaria alternata*	[67]
	LAMP	*Fusarium oxysporum* *Bipolaris sorokiniana*	[17]
